# Accelerating extrapulmonary tuberculosis diagnosis with a rapid molecular assay

**DOI:** 10.1128/spectrum.00104-25

**Published:** 2025-06-12

**Authors:** Christelle Guillet-Caruba, Caroline Bénet, Haroun Jmel, Florence Doucet-Populaire, Nadège Bourgeois-Nicolaos

**Affiliations:** 1 AP-HP Université Paris-Saclay Hôpital Antoine Béclère, Service de Bactériologie-Hygiène, Clamart, France; 2 Laboratoire de Biologie Médicale de Référence, AP-HP Université Paris-Saclay, Orsay, France; 3 I2BC, CEA, CNRS, Université Paris-Saclay 27048, Gif-sur-Yvette, Île-de-France, France; The University of Arizona, Tucson, Arizona, USA

**Keywords:** extrapulmonary tuberculosis, *Mycobacterium tuberculosis*, molecular methods, nucleic acid technology, lymph node tuberculosis, time to result, rifampin resistance, Xpert MTB/RIF Ultra

## Abstract

Extrapulmonary tuberculosis (EPTB) is characterized by a very low bacterial load and could remain undiagnosed for a long time in a number of cases due to atypical presentation. Conventional methods and their time to results are not always helpful in rapidly making an EPTB diagnosis and initiating appropriate treatment. In order to accelerate the bacteriological confirmation, we evaluated the benefits of systematically using nucleic acid amplification test (NAAT) on extrapulmonary specimens for TB diagnosis in a developed country. From November 2017 to March 2021, all extrapulmonary samples received at the mycobacteriology department were subjected to both conventional methods and Xpert MTB/RIF Ultra. The time savings and the diagnostic performance of reflex tests were evaluated. During the study period, 691 samples were included, 85 (12.3%) were NAAT positive for *Mycobacterium tuberculosis* complex (MTBC), and only 1 for a *rpoB* mutation. The overall rate of invalid NAAT results was low, 0.9%. The average time to results of NAAT was 1.3 days compared to 18 days for positive culture, allowed to accelerate the EPTB diagnosis by 16 days. Overall NAAT sensitivity and specificity were 87.9% and 94.6%, respectively. No discrepancy was found among the 67 results involving the detection of rifampicin resistance. NAAT is a quick and consistent diagnostic test for EPTB with a potential turnaround time of 2 hours. Its systematic use could allow EPTB diagnosis and treatment faster by revealing results days to weeks before culture and drug susceptibility testing.

**IMPORTANCE:**

Tuberculosis is considered the deadliest infectious disease in the world. Although tuberculosis most commonly affects the lungs, it also affects other sites referred to as extrapulmonary tuberculosis (EPTB). Diagnosis of EPTB is difficult and often delayed. We evaluated the benefits of systematically and routinely using a nucleic acid amplification test on all extrapulmonary specimens received at the mycobacterial core laboratory for EPTB diagnosis. Using a rapid (80 minutes) and easy-to-perform test, we accelerated the definitive diagnosis by an average of 16 days.

## INTRODUCTION

Tuberculosis (TB) remains a major public health problem in developed countries despite the progress made in recent years (1). In France, 4,606 cases were reported in 2020, and 1,466 (32%) were extrapulmonary tuberculosis (EPTB) (2). EPTB can affect any organ, including lymph nodes (most common), bones or joints, and genitourinary and gastrointestinal systems. These forms are not usually contagious but can cause significant morbidity and mortality (3). In recent decades, an increase in EPTB rates in developed countries has been observed. In general, EPTB affects younger subjects than do pulmonary tuberculosis (PTB) (4). In 2018, an analysis of a cohort of 1,259 patients with EPTB showed that an age of over 45 years, female gender, positive HIV status, and end-stage renal disease may be associated with EPTB (4). It is also possible to find mixed forms (PTB and EPTB) with only extrapulmonary symptoms (5).

The diagnosis of EPTB is challenging due to its multiple anatomical locations, to its diverse and unusual clinical manifestations, and difficulty in obtaining adequate specimens which are generally paucibacillary (6). The diagnosis is often delayed or even ignored and is, therefore, established at an advanced stage of the disease when complications are already present (7). Diagnosis of EPTB is based on non-respiratory specimens which may require invasive biopsy (8). These samples are generally precious, small, and few in number, which can make diagnosis difficult and may lengthen diagnosis time. As clinical symptoms are not always predictive of TB, the microbiological results are essential for differential diagnosis.

In recent years, the development of nucleic acid amplification tests (NAAT) has shown great promise in improving the accuracy and efficiency of TB diagnosis. It has been a major step forward in improving TB diagnosis (9) (10). The World Health Organization specifically recommends rapid NAAT such as Xpert MTB/RIF and Xpert MTB/RIF Ultra (Cepheid, Sunnyvale, United States of America) for more rapid diagnosis (1). These tests have a high diagnostic accuracy and lead to major improvements in the early detection of susceptible and drug-resistant forms of tuberculosis (1). However, for extrapulmonary specimens, sensitivity varies among the different NAATs. Xpert MTB/RIF Ultra assay (ULTRA) has been developed to improve the sensitivity with a detection limit of 15.6 CFU/mL of sputum (11, 12). This test is easy to use and can detect *M. tuberculosis* complex DNA and *rpoB* gene mutations associated with rifampicin resistance in less than 80 minutes (7).

The aim of this study was to evaluate the benefits of systematically and routinely using NAAT on all extrapulmonary specimens received at the mycobacterial core laboratory for EPTB diagnosis. We use the ULTRA because it is quick and easy to use. We compared it with the conventional phenotypic methods: smear examination, culture, and drug susceptibility testing (DST). The time saving of the TB confirmation with this systematic use of NAAT was evaluated.

## MATERIALS AND METHODS

### Study design and method

Over a 41-month period (from November 2017 to March 2021), all extrapulmonary samples sent from seven hospitals to the mycobacteriology laboratory of the Microbiology Department at the Paris Saclay University Hospital were tested using the ULTRA kit. We retrospectively compared the results with those of conventional microbiological methods: smear examination and culture.

### Microbiological procedures

The manufacturer does not provide technical instructions for extra-pulmonary samples. We have chosen to adapt the instructions as for pulmonary samples: whatever the type of sample, 2 mL of Xpert reagent are mixed with 1 mL of sample, avoiding visible agglomerates. Before the study, we validated this new technique on extra-pulmonary samples in accordance with the requirements of the COFRAC (the French quality control commission) and the ISO15189 quality standard.

Extrapulmonary samples (excluding urine) were processed within 24 hours except on weekends (72 hours) as follows: Auramine fluorescence smear microscopy (RAL STAINERcolorator, Fluo Ral coloration kit) followed by culture in solid Löwenstein–Jensen media (Bio-Rad) and liquid MGIT media (MGIT960, BD Diagnostic Systems) and finally Xpert MTB/RIF Ultra (Cepheid). Extrapulmonary samples were not subjected to a decontamination-fluidification step, and tissue biopsies were processed by Ultra-Turrax grinding in single-use tubes containing stainless steel balls and physiological water.

For the detection of *Mycobacterium tuberculosis* complex (MTBC), the ULTRA assay offers six semi-quantitative categories of results depending on MTBC DNA concentration: High, Medium, Low, Very Low, Trace, and Undetected.

The results of the detection of mutations associated with rifampicin resistance are divided into three qualitative categories: Not detected, Detected, and Indeterminate.

A DST was also performed for positive cultures using the phenotypic susceptibility testing SIRE (S: streptomycin, I: isoniazid, R: rifampicin, E: ethambutol) and PZA (pyrazinamide) applying the standard critical concentrations to detect resistances: 1.0 and 4.0 µg/mL for S, 0.1 and 0.4  µg/mL for I, 1.0  µg/mL for R, 5.0 and 7.5 µg/mL for E, and 100  µg/mL for PZA (BACTEC MGIT 960 SIRE Kit and BACTEC MGIT 960 PZA Kit [Becton Dickinson]).

All these standard procedures comply with the biosafety measures required for handling MTBC in a BSL-3 laboratory, in compliance with the TB laboratory biosafety manual WHO 2012 and the requirements of the COFRAC.

ULTRA performances were compared to culture and DST as reference standards. The time to results of each method was measured.

### Statistical analysis

We reported the sensitivity and specificity of ULTRA test compared with the culture results considered the reference method. We also assessed the agreement of ULTRA test for MTBC detection vs culture. Agreement percentages were calculated with the corresponding 95% CI (CI).

## RESULTS

### Characteristics of the samples

During the 41-month study period, a total of 691 extrapulmonary samples from 7 hospitals were tested for EPTB diagnosis by NAAT as well as conventional microscopy and culture at the core laboratory. The samples were divided into five groups: lymph nodes (285), serous fluid (194), bone or joint (117), pus (58), and digestive (intestine and liver) biopsy (37). Serous fluid group includes cerebrospinal fluid (CSF), ascites fluid, pleural fluid, peritoneal fluid, and other serous fluid. The pus group includes abscesses other than lymph nodes.

**Fig 1:**
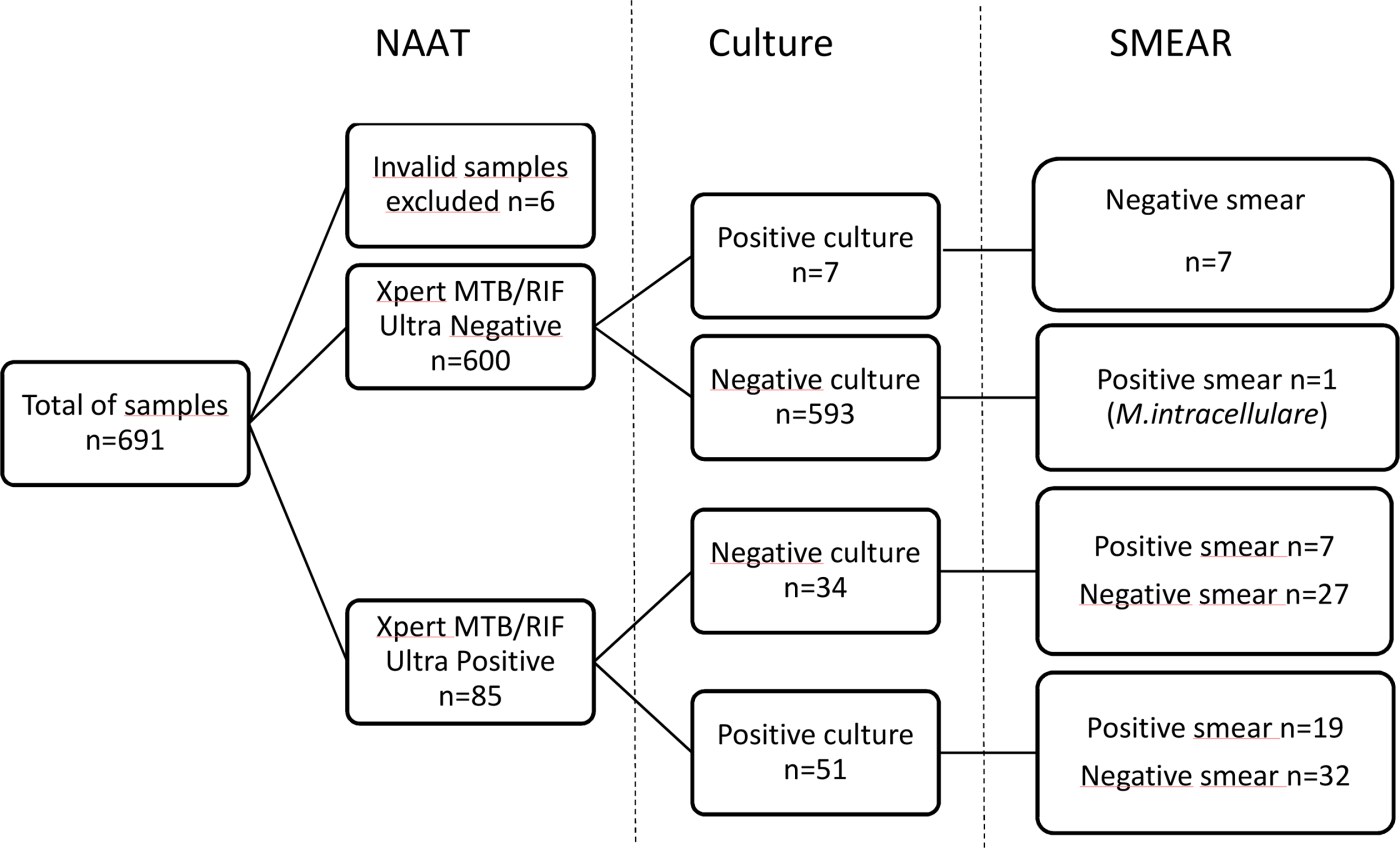
Summary of the results of the conventional methods and of NAATs for MTBC detection.

The results are listed on Fig. 1.

### NAAT results for MTBC detection

ULTRAs for the detection of MTBC were positive for 85 samples (12.3%) corresponding to these DNA concentrations: 16 Trace, 24 Very Low, 31 Low, 9 Medium, and 5 High. The overall rate of invalid results was 0.9% (6/691) corresponding to four bone or joint samples, one digestive biopsy, and one pus. None of the samples with invalid tests were positive by culture or smear. According to the type of sample, invalid results were 0/285 (0%) for lymph node; 0/194 (0%) for serous fluid, 4/117 (3.4%) for bone or joint, 1/37 (2.7%) for digestive samples, and 1/58 (1.7%) for pus.

### Microscopy results

Twenty-seven positive smears were detected among the 691 samples in our study (4%). Distribution by sample type is as follows: eight lymph nodes (2.8% or 8/285), three serous fluids (1.5% or 3/194), nine bone or joint samples (7.7% or 9/117), six pus (10.3% or 6/58), and one digestive sample (2.7% or 1/37).

### Culture results

Among the 691 samples, 58 had a positive culture (8.4%). Distribution by sample type is as follows: 22 lymph nodes (7.7% or 22/285), 5 serous fluids (2.6% or 5/194), 18 bone or joint samples (16.2% or 19/117), 11 pus (19% or 11/58), and 2 digestive samples (3% or 2/58).

### Comparison of results

We excluded the six invalid tests for the rest of the study.

#### NAAT/microscopy

Among the 85 samples with MTBC positive NAAT, the smear was positive in 26 samples (30.6%). The distribution of semi-quantitative DNA categories was as follows: 14 Low (54%), seven Medium (27%), and five High (19%).

One sample (lymph node) was ULTRA negative/smear positive and grew as *M. intracellulare*. It was considered negative for MTBC.

#### NAAT/culture

Among the 85 samples with MTBC positive NAAT, cultures were positive in 51 samples (60%).

The 34 samples with a positive NAAT result and negative culture correspond to 11 lymph nodes, 10 serous fluids, 5 pus, 7 bone or joint, and 1 digestive sample. To better understand these discrepancies between NAAT and culture, we looked for information concerning possible tuberculosis treatment on the prescription sheets. Among these 34 samples, 24 were from patients with ongoing TB treatment or who were not following the protocol correctly or who were treated immediately after the sampling. The information about a possible TB treatment was missing for the other 10 samples, but the smear was positive for 1 of these 10.

Among the 600 negative NAAT samples, 7 were positive in culture.

### Time to results of culture and NAAT

The global mean time of detection of positive culture was 18 days [range 7–44] for liquid cultures and 37 days [range 22–52] for solid culture. In liquid culture, the mean time of positivity was 21 days [range 13–30] for negative smear samples and 12 days [range 9–15] for positive smear samples.

**Fig 2:**
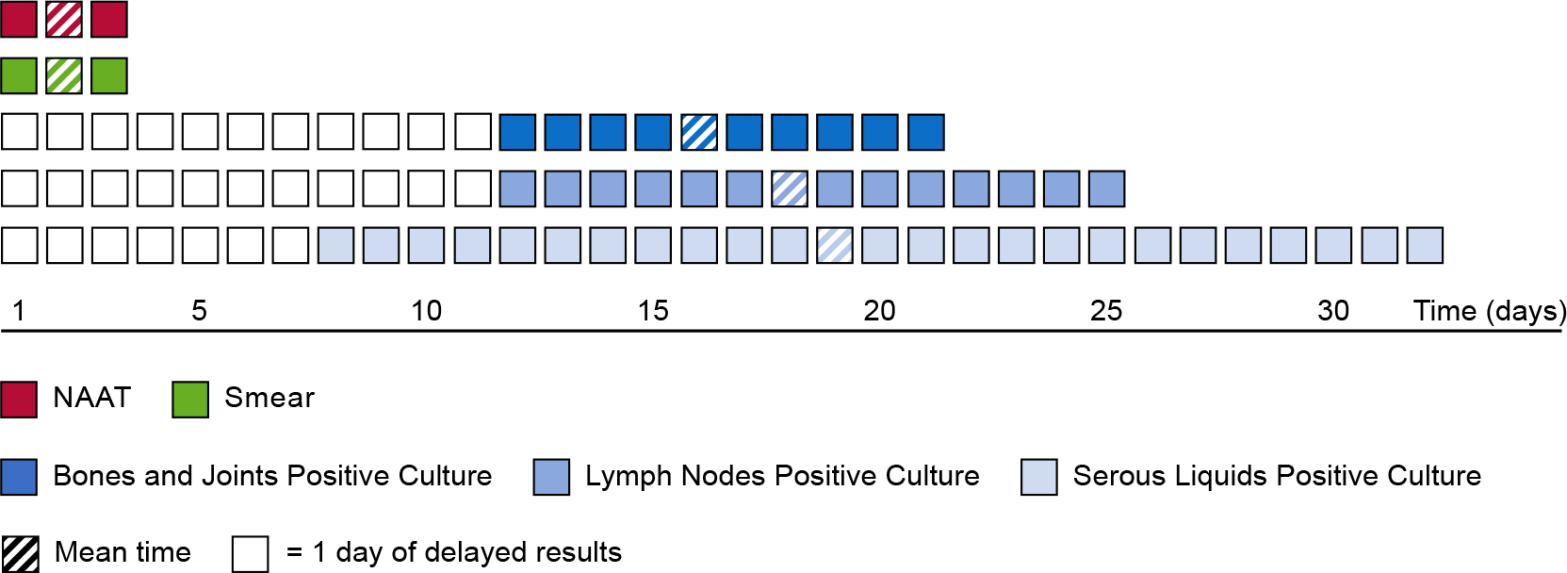
Time to positive result of NAAT, smear, and culture for three main sample categories.

Figure 2 shows the time saved using NAAT compared with culture as a gold standard for the three main categories of samples (lymph node, bones and joint, and serous fluid). During the study period, 80% of samples were received in our laboratory during working days allowing us to give the NAAT results to the physicians in less than 24 hours (1 day). For the remaining 20% of the samples, results are returned within a maximum of 72 hours (3 days). In summary, for 80% of serous liquid samples, we gained 17.6 days for the diagnosis, 16 days for lymph nodes, and 14.3 days for bones and joint samples (difference between mean time to obtain a positive liquid culture in days and 1 day to obtain NAAT result).

Overall, the decrease in the average duration of positivity is inversely proportional to the amount of DNA detected by NAAT. Concerning the time savings for diagnosis, we gained approximately 23 days for Trace results, 18 for Very Low result, 12 for Low result, and 9 for Medium and High result, respectively.

### NAAT/culture/microscopy

Among the 34 samples with positive NAAT results and negative cultures, the smear results were as follows: 7 smears were positive (NAAT results: 4 Low, 2 Medium, 1 High) and 27 were negative (NAAT results: 8 Trace, 8 Very Low, 10 Low, 1 Medium).

We obtained seven samples with negative NAAT result, negative smear, and positive culture which represents 1.2% of all samples.

Using culture as the standard of reference, overall ULTRA results for the detection of MTBC sensitivity and specificity were 87.9% (95% CI 76.7–95.0) and 94.6% (95% CI 92.5–96.2), respectively. According to the type of samples, the sensitivity and specificity vary from 33.3% to 100% and from 83.3% to 100%, respectively. Overall agreement for MTBC detection was 94.0% (95% CI 92.0–95.6), ranging from 89.5% to 97.2% depending on the types of samples (Table 1).

**TABLE 1:** NAAT performances for MTBC detection vs culture as reference standard according to the type of samples (*n* = 685)

Type of sample	Number of samples	NAAT+ /C+	NAAT+ /C−	NAAT- /C+	NAAT- /C−	Sensitivity % (CI 95%)	Specificity % (CI 95%)	Agreement % (CI 95%)
**Digestive**	36	2	1	0	33	100 (15.8–100)	97.1 (84.7–99.9)	97.2 (85.8–99.5)
**Lymph nodes**	285	19	11	3	252	86.4 (65.1–97.1)	95.8 (92.6–97.9)	95.1 (91.9–97.0)
**Bones and joint**	113	17	7	1	88	94.4 (83.8–100)	92.6 (85.3–96.9)	92.9 (86.6–96.4)
**Serous fluid total**	194	3	10	2	179	60.0 (14.6–94.7)	94.7 (90.5–97.4)	93.8 (89.5–96.4)
Cerebrospinal fluid	73	0	7	0	66	N/A	90.4 (81.2–96.1)	N/A
Other serous fluid	13	1	2	0	10	100 (2.5–100)	83.3 (51.6–97.9)	N/A
Ascites fluid	20	1	0	0	19	100 (2.5–100)	100 (84.2–100)	N/A
Pleural fluid	82	1	1	2	78	33.3 (0.84–90.5)	98.7 (93.1–99.9)	N/A
Peritoneal fluid	6	0	0	0	6	N/A	100 (54.1–100)	N/A
**Pus**	57	10	5	1	41	90.9 (58.7–99.8)	89.1 (76.4–96.4)	89.5 (78.9–95.0)
**Total**	685^*a*^	51	34	7	593	87.9 (76.7–95.0)	94.6 (92.5–96.2)	94.0 (92.0–95.6)

^*a*^ Exclusion of invalid tests; C+: MTBC positive culture; C−: MTBC negative culture; NAAT+: Positive ULTRA; NAAT−: Negative ULTRA ; N/A: non-applicable.

### Trace results

We had 16 Trace NAAT results. The samples included seven serous fluids (including five CSF, one pleural fluid, and one ascites fluid), four biopsies from spinal discs, three lymph nodes, one biopsy from hepatic tissue, and one biopsy from indeterminate tissue. All samples were smear-negative, and half were culture-positive.

### Antibiotic susceptibility

*RpoB* mutations associated with rifampicin resistance can be detected by ULTRA directly on samples. Among the 85 MTBC-ULTRA positive samples, the detection of mutations in *rpoB* was indeterminate in 17 samples, undetected in 67 samples, and detected in 1. The 17 indeterminate results correspond to 16 Trace results and 1 Low.

There was no discrepancy between the DST and the NAAT results. The one case detected with resistance to rifampicin was a multidrug-resistant strain in a CSF sample and was confirmed by the French reference centre. There were no discrepancies among the 51 usable results regarding the detection of rifampicin resistance.

## DISCUSSION

The primary aim of this study was to assess the benefits of incorporating a rapid test into the time-consuming process of extrapulmonary tuberculosis diagnosis. A secondary objective was to highlight the performance of this test on different types of specimens compared with conventional methods.

Before addressing these two objectives, we can already touch on the strengths of our study. Indeed, the first strength is the diversity and the large number of biological specimens (*n* = 691). Our recruitment corresponds to worldwide epidemiology with 27% of EPTB in our notified cases of TB during the period studied. In 2019, 15% of notified cases worldwide were extrapulmonary. Pertinent to our study, this proportion was larger (around 40%) in several high-income countries (13). Samples from lymph nodes are the major type of samples in our study which is also consistent since TB lymphadenitis is the most common form of EPTB and represents about 35% of cases (14). The second most common location of EPTB found in literature is bone or joint correlating with our sample size (15).

As already discussed, 72 hours (3 days) was the maximum time needed to deliver the NAAT results, and it concerns 20% of all samples received in our laboratory. We can estimate the number of days saved in detecting EPTB compared to the mean time of culture positivity for the main categories of samples (Fig. 2). If we focus on the global mean time of detection of positive culture of 18 days, we can deduce that NAAT accelerates the EPTB diagnosis by 16 days for 80% of patients, facilitating quick appropriate treatment and management of the disease. The time saved by using ULTRA as a point-of-care test was also demonstrated in our latest study in a French prison hospital (15). Time to microbiological diagnosis was reduced by around 18 days for 13 inmates with smear-negative sputum, avoiding the need to mobilize substantial resources (escort, transport) to take fibroscopic samples.

Despite the large sampling, we had a very low overall rate of invalid NAAT results (0.9%). There were no invalid results for serous fluid and lymph node samples in our study. This result is particularly significant because, as already mentioned, lymphadenitis is the most common form of EPTB. As in our study, other studies such as Wu et al. (16) found 9/244 (3.7%) invalid ULTRA results. Yu et al. found 2/116 (1.7%) invalid results with fine needle aspiration biopsies of lymph nodes and excluded these samples (14). Invalid results were considered and classified negative (17, 18).

Due to this negligible rate of invalid NAAT results, we were able to give a quick NAAT result for 99.1% of the samples received. The speed of our results validates the systematic use of NAAT whatever the type of samples.

In our study, the overall sensitivity of smear microscopy was 44.8% (95% CI [32.03%; 57.63%]), and the overall sensitivity of ULTRA was 87.9% (95% CI [76.7%; 95.0%]). These two techniques have an identical turnaround time: a maximum of 72 hours but do not have the same sensitivity. In addition, unlike ULTRA which is easy to reproduce, the screening of smears depends on the experience of the operator. This would make ULTRA a decisive tool in rapid diagnosis. However, it is necessary to emphasize that NAAT should not replace smear and culture as stressed in the WHO consolidated guidelines on tuberculosis which recommended using NAAT to help diagnose EPTB in symptomatic adults and children in association with smear microscopy and culture (1). In our study, seven samples were NAAT negative and culture positive. This is explained by the difference in the limit of detection (LOD) between culture and ULTRA. The culture LOD is 1 CFU/mL, whereas that of Ultra is 15 CFU/mL (11).

Concerning the ULTRA sensitivity performances on the different types of sampling, the mediocre value of pleural fluid (33%) could not be taken into account due to the low proportion (3.6%) of positive cultures among the 82 samples. The sensitivity found in the meta-analysis of Gong et al. (19) is closer to ours (30%), which may suggest that this test is not as effective for pleural fluid as for other types of samples. The Cochrane review of 2020 (8) estimated pooled sensitivity at 75% (95% CI 58 to 86.4). In the studies included, sensitivity varied from 65% to 100% (16, 20–22).

Sensitivity performances for the other types of specimens are satisfactory in our study, even if it is a little lower for lymph nodes than for the other extra pulmonary specimens (86.4%), unlike Nasrin et al. who found that lymph nodes were the type of sample for which sensitivity was the highest (23). Our findings are still similar to the value retained by Cochrane (8). One explanation for this less efficient performance puts forward by Cochrane pinpoints the greater proportion of patients probably already treated when the clinical presentation is lymph node. Indeed, as already mentioned, lymph node forms are the most frequent forms of EPTB. Clinical diagnosis is, therefore, carried out more frequently and more easily by physicians who do not always wait for sampling to treat. Even if we do not know what proportion of patients were treated before sampling in our group, this assumption could explain the lower sensitivity performance.

The overall specificity of ULTRA was 94.6% (95% CI [92.5%; 96.2%]). If we analyze our results by type of sample, we can observe that specificity is satisfactory for every type of sample: the best specificity being 97.1% for digestive samples and the lowest being 89.1% for pus. These values are more substantial for lymph nodes, bone and joint samples, and serous fluid since the sample numbers are larger (respectively, 285, 113, and 194). This observation suggests that a negative NAAT result can be taken seriously even if it is necessary to wait for the culture result to validate an unquestionable negative. Moreover, we are in concordance with other studies (16, 17, 20, 24) included in the meta-analysis of Zhang et al. (25), in which sensitivity varies from 70% to 91% and specificity from 92% to 100% for extrapulmonary samples. We also agree with Hoel et al. (13) who found a specificity of 99% as well as with the recent meta-analysis of Gong et al. (19) with their specificity at 98%.

Timely detection of causative pathogens and their antimicrobial resistance is essential to guiding targeted therapies. This makes the ULTRA particularly useful when the smear is negative.

We obtained 16 samples with NAAT results at Trace. These results do not seem to correlate with the type of samples. All samples were smear negative, and eight (50%) were culture negative. These eight samples were from patients already treated. All these 16 patients were considered by clinicians as infected patients, so we considered a Trace result as positive even if the smear and culture were negative. Dowling et al. (26) conducted a study on pulmonary and extra pulmonary Trace results where cultures were positive in 37/71 (52%). Wu et al. had 2/17 (11.8%) ULTRA positive with negative culture in fine needle aspiration samples and 18/39 (46%) ULTRA positive with culture negative in tissue samples. They considered all these results to be positive (14). Guillouzouic et al. argued that considering Trace results as positive allowed early diagnosis of paucibacillary tuberculosis (especially paediatric and extrapulmonary forms). This test also allowed 87% of severe cases to be treated within 1–7 days of the Trace result in their study (27). The WHO has recently issued recommendations regarding the detection of Trace in cerebrospinal fluid, lymph nodes, and tissues. Trace results should be considered a bacteriological confirmation of TB and patients should be treated with first-line TB drugs unless they are at high risk of having a multidrug-resistant strain (27). This recommendation includes the fact that the main limitation of these Trace results is the lack of detection of rifampicin resistance due to the very low levels of MTBC DNA.

However, this result must be compared according to the WHO consolidated guidelines on tuberculosis (2) to clinical presentation and patient context before initiating treatment.

Concerning discrepant results, there were 27 ULTRA positive samples with negative cultures and smears (8 Trace, 8 Very Low, 10 Low, 1 Medium).

To explain these differences, first, it is established that ULTRA cannot distinguish viable from dead bacilli, and NAAT tests are known to be less sensitive than culture (28).

Second, we can cite a recent article which attempted to correlate the quantity of bacilli and the performances of culture and NAAT on respiratory samples. They highlighted that with a late cycle threshold and, therefore, a small quantity of DNA, there are significant differences between the performances of cultures and that of NAAT without attributing better performance to either of the techniques (29). However, the limit of detection for ULTRA on extrapulmonary specimens is not published.

In short, the lower the quantity of bacteria, the more likely to be discrepancies in the results of different techniques. In our study, which focused exclusively on extrapulmonary samples, 26/27 NAAT results showed discrepancies due to low quantification of DNA (Low, Very Low, and Trace).

Concerning the significant differences in culture positivity between Trace vs High and Trace vs Medium results samples, we agree with Perez-Risco et al. (20) who found a significant difference in time required for liquid culture growth in smear negative samples: Trace vs Medium (*P* = 0.003). According to our results, the time to reach positivity of liquid cultures varies depending on the semi-quantitative result of the NAAT. Our results show that the mean time to culture positivity seems to be inversely proportional to the semi-quantitative MTBC DNA estimation given by the ULTRA. Moreover, the correlation between the semi-quantitative results and bacilli detection has been demonstrated (30, 31). The higher the NAAT result and the richer the sample in bacilli, the greater the probability of TB transmission. For this reason, the NAAT result, and its semi-quantitative category could be used to evaluate and predict this risk of transmitting infection.

### Performances for the detection of rifampicin resistance

Concerning performances in the detection of rifampicin resistance, sensitivity, and specificity were both 100% true. One limitation of our study is that we had only one rifampicin- resistant specimen, which is, nevertheless, in agreement with the low incidence of multidrug-resistant tuberculosis in France (2). According to Cochrane 2020 (8), ULTRA pooled sensitivity and specificity for detection of rifampicin resistance in EPTB were 100%. In other words, the result for rifampicin resistance could be considered an essential element in determining treatment. However, in the case of trace or very low NAAT results, indicating the poor presence of MTBC DNA, ULTRA has the limitation of not being able to detect the single copy of the *rpoB* gene fragment containing the region conferring rifampicin resistance and provides an uninterpretable result (27).

### Conclusion

Although the performance of the assay varies depending on the type of extrapulmonary specimens, implementing NAAT in the EPTB diagnosis algorithm accelerates and optimizes a reliable microbiological diagnosis. Although PCR is a less sensitive method than culture, the result is obtained more quickly, which allows treatment to be started early and preventive measures put in place. Associating a culture with NAAT is always recommended in order to isolate a strain, to confirm the diagnosis, study susceptibility to antibiotics, and carry out other microbiological or epidemiological studies.
